# An observational cohort study of body mass index in pediatric vitiligo

**DOI:** 10.1097/JW9.0000000000000177

**Published:** 2024-09-18

**Authors:** Ross O’Hagan, Shira Wieder, Marcel Perl, Jonathan I. Silverberg, Nanette B. Silverberg

**Affiliations:** a Department of Dermatology, Icahn School of Medicine at Mount Sinai, New York City, New York; b George Washington University School of Medicine and Health Sciences, Washington, District of Columbia

**Keywords:** BMI, BSA, metabolic syndrome, overweight, vitiligo

**What is known about this subject in regard to women and their families?**

- Half of vitiligo cases begin in childhood, before the age of 20 years, with more than half of these cases occurring in girls.
- Vitiligo can cause anxiety and depression in patients and their parents, especially female caregivers.

**What is new from this article as messages for women and their families?**

- While body mass index (BMI) has been notably associated with vitiligo distribution in adults, there appears to be no effect on the disease in children.
- The trends toward higher BMI with age suggest that weight gain is associated with age in vitiligo, which may later play a role in adult health.

Vitiligo is an autoimmune pathology affecting around 1.52% of children aged 4 to 11 years and 2.16% of adolescents aged 12 to 17 years in the U.S. population.^1^ Previous work demonstrated that overweight and obese adult patients with vitiligo had more lesions over sites of friction on the abdomen and axillae,^2^ and there is no literature exploring birthweight, body mass index (BMI), and pediatric vitiligo. This study explores the relationship between birthweight, reported BMI classifications, and pediatric vitiligo characteristics.

A total of 232 parent/child dyads members of an online support group volunteered for an IRB-exempt survey where they self-reported their children’s data (refer to methodology in Silverberg et al.^3^). Parents reported their children’s demographic information, height, and weight, as well as their vitiligo distribution and birthweight.^3–5^ Analysis was performed with R (version 1.4, Free Software Foundation, Boston, MA). To calculate the BMIs, the R library “PAutilities: Streamline Physical Activity Research” was used. BMI classifications were underweight for patients with a BMI greater than 18.5, normal for patients with a BMI equal to 18.5 or larger but less than 25, and overweight for patients with a BMI equal to or over 25, and for some analyses, obese for BMI greater than 30 kg/m^2^.

There was a trend of decreasing median age of onset with increasing birthweight (Fig. 1), but this was not statistically significant when we grouped the patients by BMI class (Supplementary Table 1, http://links.lww.com/IJWD/A54). There is a trend toward older age for children with higher BMI classes (*P* = .06), supporting increasing BMI with age in pediatric vitiligo patients. The mouth was more common for underweight children (*P* = .033). There was a trend toward earlier onset of vitiligo with increasing birthweight, without statistical significance.

**Fig. 1.**
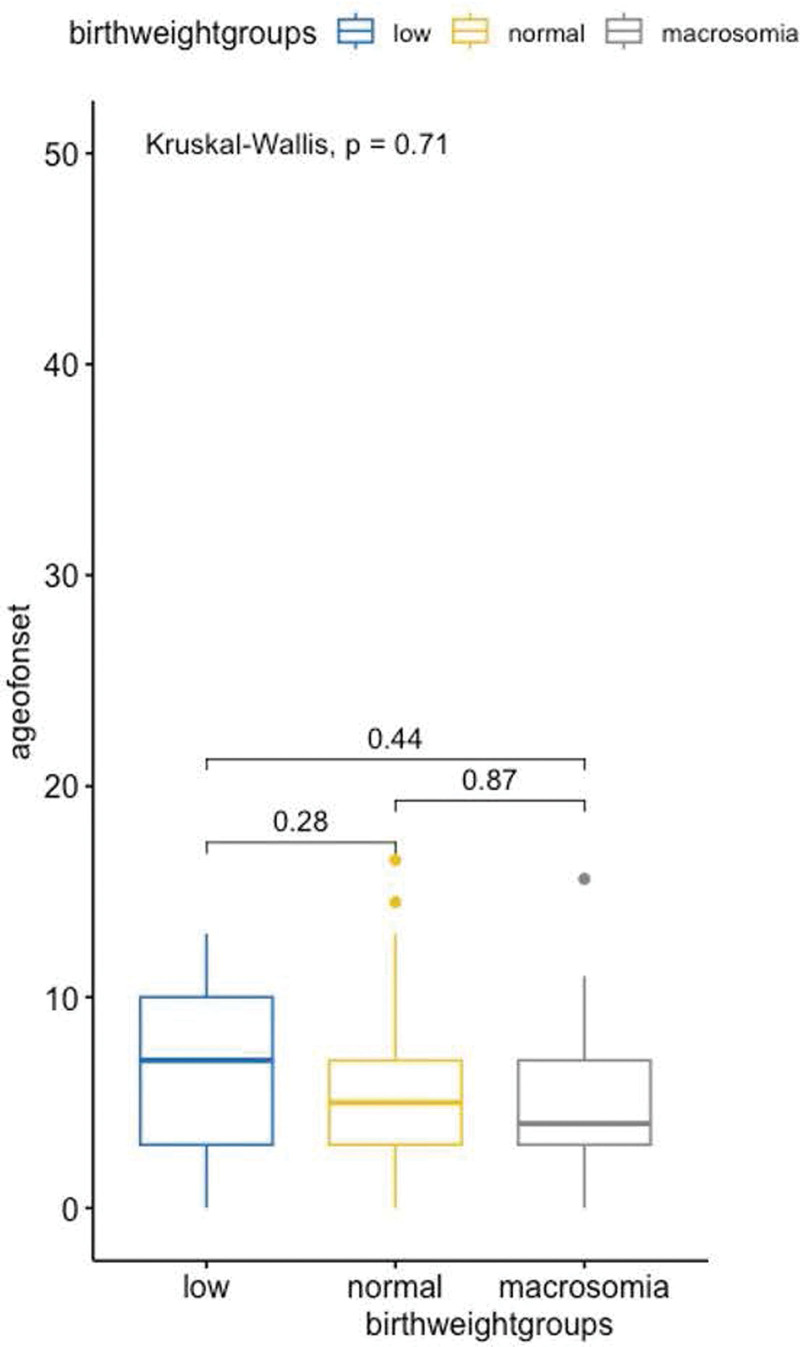
Association of birthweight and age of vitiligo onset.

There is a positive trend in locations and age (Table 1) when looked at for self-reported body surface area (BSA) of 1–25% versus >25% (*P* = .044). Wrists, hands, hips, legs, ankles, and feet were statistically more likely to be involved by 14–17 years and over 0–6 years (*P* < 0.05 for each site). BSA was 1–25% in 79% of kids under 12 years of age and 75% for kids >12 years of age. For those with a BSA of 26%+, all anatomical regions explored were statistically significant (*P* < .05) other than the specific locations of the scalp, eyelids, lips, or mouth.

**Table 1:** Vitiligo characteristics by age group

Variable	*N*	0–6 y, *N* = 66^a^	6–12 y, *N* = 103^a^	12–14 y, *N* = 27^a^	14–17 y, *N* = 36^a^	*P* value^b^
Age	232					<.001
*N*		66	103	27	36	
Mean (SD)		4.6 (1.4)	9.6 (1.7)	13.3 (0.5)	16.0 (0.9)	
Median (IQR)		5.0 (4.0–6.0)	10.0 (8.0–11.0)	13.0 (13.0–14.0)	16.0 (15.0–17.0)	
Range		0.5–6.0	7.0–12.0	13.0–14.0	15.0–17.0	
Duration of disease	227					<.001
*N*		64	102	27	34	
Mean (SD)		1.3 (1.2)	4.0 (3.0)	6.6 (3.5)	7.5 (4.5)	
Median (IQR)		0.9 (0.5–2.0)	4.0 (1.0–6.0)	7.0 (4.2–9.0)	7.0 (4.0–10.0)	
Range		0.0–5.0	0.0–11.0	0.5–13.0	0.0–16.0	
What is your child’s sex (male or female)?	232					.2
Female		45 (68%)	56 (54%)	13 (48%)	21 (58%)	
Male		21 (32%)	47 (46%)	14 (52%)	15 (42%)	
How do you describe your child?	209					.041
African-American		3 (5.2%)	4 (4.4%)	0 (0%)	1 (2.9%)	
Asian		3 (5.2%)	5 (5.5%)	0 (0%)	0 (0%)	
Caucasian		27 (47%)	61 (67%)	20 (77%)	29 (85%)	
Hispanic		6 (10%)	11 (12%)	3 (12%)	3 (8.8%)	
Indian		15 (26%)	8 (8.8%)	2 (7.7%)	1 (2.9%)	
Southeast Asian		4 (6.9%)	2 (2.2%)	1 (3.8%)	0 (0%)	
How much body surface area does the vitiligo affect?	231					.2
1–25%		59 (91%)	74 (72%)	21 (78%)	26 (72%)	
100% of the body		0 (0%)	1 (1.0%)	0 (0%)	1 (2.8%)	
26–50%		5 (7.7%)	18 (17%)	3 (11%)	6 (17%)	
51–75%		1 (1.5%)	6 (5.8%)	2 (7.4%)	2 (5.6%)	
76–99%		0 (0%)	4 (3.9%)	1 (3.7%)	1 (2.8%)	
Scalp	173	16 (38%)	17 (21%)	3 (16%)	11 (35%)	.10
Gray hair	174	15 (35%)	16 (20%)	6 (32%)	10 (33%)	.2
Eyelids	189	29 (55%)	51 (61%)	11 (55%)	15 (47%)	.6
Lips	173	12 (28%)	22 (26%)	6 (35%)	8 (28%)	.9
Mouth	166	4 (9.8%)	9 (11%)	0 (0%)	1 (3.8%)	.5
Chest	179	24 (51%)	45 (55%)	9 (47%)	17 (55%)	>.9
Stomach	179	22 (47%)	50 (60%)	13 (72%)	16 (53%)	.3
Back	177	23 (49%)	47 (59%)	7 (35%)	18 (60%)	.2
Axillae	176	21 (45%)	49 (59%)	9 (53%)	14 (48%)	.4
Arms	166	10 (26%)	39 (49%)	8 (47%)	14 (45%)	.10
Elbows	166	12 (31%)	41 (53%)	10 (56%)	17 (55%)	.10
Wrists	165	10 (24%)	32 (42%)	10 (56%)	16 (53%)	.037
Hands	163	8 (21%)	34 (44%)	9 (50%)	15 (50%)	.037
Fingers	172	14 (33%)	39 (49%)	11 (58%)	18 (58%)	.12
Hips	166	14 (34%)	45 (58%)	8 (47%)	21 (70%)	.015
Genitals	174	14 (32%)	33 (41%)	9 (47%)	16 (52%)	.3
Buttocks	162	10 (24%)	33 (44%)	6 (35%)	10 (34%)	.2
Legs	185	20 (42%)	55 (65%)	15 (71%)	19 (59%)	.038
Knees	188	25 (54%)	59 (69%)	18 (82%)	26 (76%)	.077
Ankles	172	17 (39%)	52 (66%)	16 (84%)	19 (63%)	.004
Feet	175	16 (36%)	49 (60%)	13 (72%)	16 (52%)	.018
Toes	167	13 (31%)	34 (42%)	6 (40%)	13 (43%)	.6
Body mass index	200					.12
1 (underweight)		13 (27%)	13 (14%)	6 (23%)	2 (5.9%)	
2 (normal weight)		22 (45%)	53 (58%)	16 (62%)	24 (71%)	
3 (over weight/obese)		14 (29%)	25 (27%)	4 (15%)	8 (24%)	

a *n* (%).

b Kruskal–Wallis rank sum test; Fisher exact test for count data with simulated *P* value (based on 2000 replicates).

When we explore BMI and relationship in a pediatric cohort, we see there is no significant relationship, except for oral vitiligo being more common in underweight children. This suggests that the role of clothing friction and metabolic syndrome on disease extent may be more contributory to onset than progression; however, given that the trend was not statistically significant, no conclusions can be made.

To the best of our knowledge, the impact of birthweight on pediatric vitiligo has not been explored. When we explore the impact of birth weight, we see there are some potential slight trends that may require larger studies to elucidate, specifically earlier onset of vitiligo.

A limitation of this work is that its survey format may lead to people only listing regions that are truly bothering them. Another limitation is that the cohort does not address long-term weight fluctuations. The size of the cohort was quite a bit smaller than the adult cohort, which suggests that the effect may only be seen in a much larger cohort.

Our article is the first of its kind to explore the impact of birth weight on pediatric vitiligo and explore the relationship between age and BMI on vitiligo sites of involvement. Further studies are needed to better understand how BMI and age impact the relationship between vitiligo and its sites of presentation over time.

## Conflicts of interest

The authors made the following disclosures: N.B.S. has been an advisor or received honoraria from Amryt, Incyte, Lilly, Regeneron/ Sanofi, and Verrica Pharmaceuticals. J.I.S. has received honoraria as a consultant and/or advisory board member for Abbvie, AObiome, Arcutis, Alamar, Amgen, Arena, Arcutis, Asana, Aslan, BioMX, Biosion, Bodewell, Boehringer-Ingelheim, Cara, Castle Biosciences, Celgene, Connect Biopharma, Dermavant, Dermira, Dermtech, Eli Lilly, Galderma, GlaxoSmithKline, Incyte, Kiniksa, Leo Pharma, Menlo, Novartis, Optum, Pfizer, RAPT, Regeneron, Sanofi-Genzyme, Shaperon, Union; speaker for Abbvie, Eli Lilly, Leo Pharma, Pfizer, Regeneron, Sanofi-Genzyme; institution received grants from Galderma, Pfizer. The other authors have no conflicts of interest to disclose.

## Funding

None.

## Study approval

N/A

## Author contributions

ROH performed the statistical analysis and created the tables and initial draft. MP and SW revised the dataset for analysis. JIS oversaw statistical design and revised the manuscript. NBS was the lead researcher on the study and responsible for data collection and design of the study, as well as revision of the manuscript.

## Supplementary data

Supplementary material associated with this article can be found at http://links.lww.com/IJWD/A54.

## Supplementary Material


