# Supplementary material for: An observational cohort study of body mass index in pediatric vitiligo
Source: Int J Womens Dermatol. 2024 Sep 18;10(3):e177. doi: 10.1097/JW9.0000000000000177 (PMC11410332; doi:10.1097/JW9.0000000000000177)
Supplement: Supplementary file 1 [file jw9-10-e177-s001.pdf]

Supplemental Table 1: Cohort Characteristics of the Vitiligo Patients by BMI Class

Variable	N	underweight, N = 51	normal, N = 820	overweight, N = 470	obese, N = 344	p-value[1]
**What is your sex?**	1,685					<0.001
Female		38 (75%)	618 (75%)	298 (63%)	266 (77%)	
Male		13 (25%)	202 (25%)	172 (37%)	78 (23%)	
**Age**	1,685					<0.001
Mean (SD)		31 (11)	40 (14)	45 (13)	46 (12)	
**BMI**	1,685					<0.001
Mean (SD)		16.9 (0.7)	21.9 (1.8)	27.2 (1.4)	34.2 (3.5)	
**Race**	1,685					<0.001
African-American		1 (2.0%)	19 (2.3%)	32 (6.8%)	40 (12%)	
Asian		5 (9.8%)	31 (3.8%)	13 (2.8%)	4 (1.2%)	
Caucasian		24 (47%)	536 (65%)	307 (65%)	214 (62%)	
Hispanic		2 (3.9%)	72 (8.8%)	54 (11%)	60 (17%)	
Indian		9 (18%)	76 (9.3%)	29 (6.2%)	10 (2.9%)	
other/unknown		10 (20%)	86 (10%)	35 (7.4%)	16 (4.7%)	
**BSA**	1,677					0.001
1-25%		34 (67%)	506 (62%)	273 (58%)	160 (47%)	
26-50%		11 (22%)	167 (20%)	105 (22%)	84 (25%)	
51-75%		5 (9.8%)	77 (9.4%)	41 (8.8%)	51 (15%)	
76-99%		1 (2.0%)	56 (6.8%)	38 (8.1%)	35 (10%)	
100% of the body		0 (0%)	12 (1.5%)	10 (2.1%)	11 (3.2%)	
